# Association between body mass index and happiness among young adults in Taiwan: a cross-sectional study

**DOI:** 10.3389/fpubh.2025.1566383

**Published:** 2025-07-09

**Authors:** Chi-Fang Lin, Yan-Jhu Su, Hsiao-Fang Kao, Po-Fu Lee, Mei-Ling Chao, Ding-Peng Yeh, Jiann-Cherng Shieh, Chien-Chang Ho

**Affiliations:** ^1^Department of Physical Education and Sport Sciences, National Taiwan Normal University, Taipei City, Taiwan; ^2^Office of Physical Education, Fu Jen Catholic University, New Taipei City, Taiwan; ^3^Department of Gerontology, University of Massachusetts Boston, Boston, MA, United States; ^4^Department of Sports Management, Minghsin University of Science and Technology, Hsinchu County, Taiwan; ^5^Department of Leisure Industry and Health Promotion, National Ilan University, Yilan County, Taiwan; ^6^College of Humanities and Management, National Ilan University, Yilan County, Taiwan; ^7^Department of Nursing, Meiho University, Pingtung County, Taiwan; ^8^Department of Sport Management, Aletheia University, New Taipei City, Taiwan; ^9^Graduate Institute of Library and Information Studies, National Taiwan Normal University, Taipei City, Taiwan; ^10^Department of Physical Education, Fu Jen Catholic University, New Taipei City, Taiwan; ^11^Sports Medicine Center, Fu Jen Catholic Hospital, New Taipei City, Taiwan

**Keywords:** adiposity, cross-sectional study, subjective wellbeing, Taiwan, young adult

## Abstract

**Background:**

Body mass index (BMI) is widely used as an indicator of physical health and has been associated with various psychological and social outcomes. While previous studies have explored the link between BMI and mental wellbeing, findings remain inconsistent, especially among young adults. This study aimed to examine the associations between BMI and self-reported happiness among young adults in Taiwan.

**Methods:**

A cross-sectional study was conducted using secondary datasets from the Taiwan National Physical Activity Survey, a nationally representative survey of the Taiwanese population. A total of 10,638 young adults aged 18–44 years were enrolled in this study between August and October 2020. Demographic characteristics, self-reported health status, self-evaluations (comprising height, body weight, BMI, and happiness levels), and residence zip codes were among the data obtained through the national telephone survey.

**Results:**

The results revealed that, participants in very happy (*β* = 3.167, *p* < 0.001), happy (*β* = 3.239, *p* < 0.001), fair (*β* = 3.058, *p* < 0.001), and unhappy (*β* = 2.942, *p* < 0.001) groups exhibited a significantly higher BMI than did participants in very unhappy group. After adjusting for potential confounders, the very happy, happy, fair, and unhappy groups showed statistically associated with underweight risk reduction (OR = 0.196, 95% CI: 0.061–0.633; OR = 0.258, 95% CI: 0.085–0.785; OR = 0.271, 95% CI: 0.083–0.882; OR = 0.222, 95% CI: 0.069–0.714).

**Conclusion:**

Overall, this study revealed that happiness levels were positively associated with BMI, and the increments of happiness levels may have reducing the risk of underweight occurrence.

## 1 Introduction

In a 2020 report, the World Health Organization (WHO) revealed that more than 1.9 billion adults worldwide were overweight, of whom, over 650 million were obese (1). Obesity has become a global epidemic and is undoubtedly related to an increased risk of cardiovascular disease (CVD), hypertension, diabetes, osteoarthritis, and cancer, reducing the quality of life and increasing the risk of premature death (2, 3). Notably, Taiwan has a significantly obese population, with 50.7% of adults classified as overweight (26.8%) and obese (23.9%) according to the 2017–2020 Taiwan Nutrition and Health Survey (4). Therefore, Taiwan should prioritize the successful prediction of future risk for overweight and obesity and ensure that effective weight management measures are followed. Body mass index (BMI) is widely used as an indicator of obesity status in adults, with the WHO setting the cut-off points at >25 kg/m^2^ for overweight and >30 kg/m^2^ for obesity. A previous study revealed that BMI is a common predictor of multiple health outcomes such as heart disease, diabetes, osteoarthritis, and anxiety disorders (5). Despite these limitations, BMI is still used in many studies due to its convenience.

Recent studies have suggested that poor mental health significantly contributes to the global burden of disease, while also impacting happiness (6). Definitions of happiness generally encompass positive mental or emotional states and satisfaction (7, 8). Happiness is now a communal health priority in several countries worldwide, as witnessed by the publication of a WHO report, which suggest that countries, including the United Kingdom, France, and Canada, pay attention to happiness indices to complement existing measures of population development, such as gross domestic product (GDP) (9). Several articles by economists and psychologists have endorsed the value of happiness (10–13). Additionally, some variables related to happiness, such as genetics, education, socioeconomic status, social networks, time use, activities, stress, marital status, family, and personality, have been confirmed in prior research (14–16).

Numerous studies have documented the association between overweight/obesity and various physical and psychological health outcomes (17). In addition to its well-known health implications, obesity has also been linked to socioeconomic factors such as education, marital status, and employment (18–23). Moreover, studies have found an association between weight loss and mental health/quality of life (24). On the other hand, the “jolly fat hypothesis” proposed by Crip and McGuinness (25) suggested that overweight or obesity was negatively related to negative mood (depression). While some studies have explored the relationship between obesity and subjective wellbeing or happiness, the evidence remains inconclusive. Some studies have reported a negative association between higher BMI and happiness or life satisfaction (26), while others have found null or different relationships among different social-demographic factors (27). These inconsistencies may stem from differences in population demographics, cultural norms, gender roles, BMI categorization, and the operationalization of “happiness” itself. For instance, some studies focus on evaluative wellbeing (e.g., life satisfaction) while others examine affective states (e.g., happiness or emotional balance), which are conceptually distinct and may yield different findings (28, 29).

Although previous analyses from several countries have revealed that physical fitness seems to be more strongly associated with mental health than other domains, these studies did not assess the composition of happiness (30–32). Furthermore, at different body component levels, limited evidence and mechanisms exist to describe the relationship between BMI and happiness. Hence, based on previous studies, our aim is to examine the associations between BMI and happiness.

Moreover, existing research has predominantly concentrated on Western contexts. To our knowledge, no prior studies have examined the association between BMI and self-reported happiness specifically among young adults in Taiwan. This population is particularly important given the unique sociocultural factors in Taiwan—such as collectivist values, body image ideals, and academic pressures—that may shape how weight status influences emotional wellbeing (33). Young adulthood is also a critical developmental stage where identity formation, social comparison, and emotional regulation interact closely with body-related perceptions.

While our study uses cross-sectional data, it offers valuable evidence in an understudied cultural context. Cross-sectional analyses remain important for identifying population-specific associations and generating hypotheses for future longitudinal research, especially where no prior local data exist. Therefore, this study aims to examine the relationship between BMI and self-reported happiness in a nationally representative sample of young adults in Taiwan. By focusing on a distinct age group and cultural setting, we contribute to a more nuanced understanding of the links between physical and emotional health.

## 2 Materials and methods

### 2.1 Study design and participants

This study employed a cross-sectional design, drawing on nationally representative survey data from the National Physical Activity Survey (TNPAS), which focuses on young adults aged 18–44 years. Young adulthood was defined as ages 18–44, consistent with Erikson’s psychosocial theory of development, which characterizes this period as a time of identity formation and pursuit of intimacy (34, 35). The Sports Administration of Taiwan’s Ministry of Education conducted the survey. The participants were selected using a random-digit dialing method, with additional procedural details provided in other sections (36, 37). The respondents comprised individuals aged 13 years or older at the time of the survey, and the sample was stratified across 22 cities and counties in Taiwan. The sample size for each city or county was determined based on its proportion of Taiwan’s total population. In 2020, the survey included 25,526 participants, achieving a sampling error of no more than 5% and a 95% confidence interval (CI) to ensure adequate statistical power. A total of 10,638 participants aged 18–44 years were included in this study, which adhered to the Declaration of Helsinki and complied with ethical standards approved by the Institutional Review Board of Fu Jen Catholic University, Taiwan (FJU-IRB C110113). Verbal consent was obtained from the participants prior to the CATIs.

### 2.2 Data collection

Data were collected through computer-assisted telephone interviews (CATIs) between August and October 2020. Highly trained and experienced interviewers facilitated the CATI process to ensure data quality. The survey gathered information on sociodemographic characteristics (age, sex, education, and occupation), leisure-time physical activity (LTPA) behaviors, self-reported health status, self-evaluations (including height, body weight, and happiness), and residence zip codes.

The sociodemographic characteristics included age, sex, education, occupation, and urbanization of the residential place. Additionally, the study recorded variables such as participants’ LTPA behaviors, self-reported health status, self-assessments of anthropometric measures (height and weight), and happiness. The participants were divided into five age groups: 18–24, 25–29, 30–34, 35–39, and 40–44 years. Education was categorized into three levels: elementary school or lower, junior or senior high school, and college or higher. Occupations were classified into 11 categories: white collar, government servant, blue collar, owner/manager, specialist, student, housewife, retired, freelancer, jobless, and others. Urbanization of the residential place were classified into 2 categories: urban and rural. The urban category consists of schoolchildren whose residential places are located in the six largest cities such as Taipei, New Taipei, Taoyuan, Taichung, Tainan, and Kaohsiung in Taiwan. LTPA behaviors were categorized into two levels: regular LTPA (150–300 min of moderate intensity or 75–150 min of high-intensity physical activity per week) and non-regular LTPA. Self-reported health status was classified into three categories: excellent or good, fair, and very poor or poor.

### 2.3 Anthropometric variables and obesity status

Data on anthropometric variables, including height and weight, were collected through the CATI survey to calculate participants’ BMI (kg/m^2^). Based on the guidelines of the Health Promotion Administration, Ministry of Health and Welfare, Taiwan, BMI categories were defined as follows: underweight (BMI < 18.5 kg/m^2^), normal weight (BMI 18.5–24 kg/m^2^), overweight (BMI 24–27 kg/m^2^), and obese (BMI ≥ 27 kg/m^2^) (38).

### 2.4 Happiness measures

Happiness, a multidimensional concept with various determinants (39–42), has been assessed using instruments such as the Oxford Happiness Questionnaire (43) and the Life Satisfaction Scale (44). However, a single-item question (“Generally, how happy would you say you are?”) has demonstrated validity (45, 46) and high temporal stability (test–retest reliability: *r* = 0.86) (46) in measuring happiness levels. In the 2020 TNPAS survey, happiness levels were assessed using this question, rated on a 5-point Likert scale, where 1 = very unhappy, 2 = unhappy, 3 = fair, 4 = happy, and 5 = very happy.

### 2.5 Statistical analysis

Data analysis was performed using SAS 9.4 (SAS Institute, Cary, NC, USA). Descriptive statistics examined levels of happiness across various sociodemographic characteristics, anthropometric variables, and obesity status. Continuous variables were analyzed using analysis of variance (ANOVA), while categorical variables were assessed using chi-square tests. The Shapiro–Wilk test was used to evaluate the normality of data distribution.

The association between happiness and BMI was examined using multiple linear regression, with BMI as the dependent variable and adjustments made for potential confounders. Additionally, unconditional logistic regression models were employed to calculate the adjusted odds ratios (ORs) with 95% confidence intervals (CIs) for the likelihood of being obese, overweight, or underweight according to happiness levels. The results were presented as mean ± standard deviation or percentages, with statistical significance determined at *p* < 0.05 in two-tailed tests.

## 3 Results

The demographic characteristics of the study population are presented in Table 1. All participants were divided into five groups according to their happiness levels: very happy, happy, fair, unhappy, and very unhappy. Significant differences were found among the levels in education, occupation, self-reported health status, urbanization of the residential place, and regular LTPA (*p* < 0.05).

**Table 1:** Characteristics of the study participants by happiness levels in Taiwanese young adults.

Variables	Very happy (*n* = 730)	Happy (*n* = 8,246)	Fair (*n* = 643)	Unhappy (*n* = 795)	Very unhappy (*n* = 50)	*p*
Age group (%)						<0.0001*
18–24 years	32.42	23.35	14.91	11.01	30.60	
25–29 years	13.57	18.09	18.83	15.47	14.29	
30–34 years	19.27	16.75	18.98	20.67	14.29	
35–39 years	17.37	20.56	19.88	27.35	22.45	
40–44 years	17.37	21.25	27.40	25.50	18.37	
Gender (%)						0.013*
Women	44.31	48.00	47.44	42.95	36.73	
Men	55.69	52.00	52.56	57.05	63.27	
Height (cm)	167.34 ± 8.64	166.51 ± 8.48	166.94 ± 8.79	166.73 ± 8.69	166.93 ± 11.91	0.111
Body weight (kg)	64.43 ± 13.92	64.27 ± 13.59	65.11 ± 16.23	66.22 ± 15.09	57.67 ± 12.66	<0.0001*
Education (%)						<0.0001*
Elementary school or lower	0.82	0.13	0.61	0.62	0.00	
Junior or senior school	5.18	10.33	16.29	13.01	12.00	
College or higher	94.00	89.54	83.10	86.37	88.00	
Occupation (%)						<0.0001*
White collar	20.33	25.33	19.46	24.00	6.00	
Government servant	5.46	7.23	3.32	6.34	4.00	
Blue collar	21.42	20.68	32.73	26.11	10.00	
Owner/manager	5.05	4.37	1.82	6.84	8.00	
Specialists	6.28	10.52	7.99	9.95	28.00	
Student	23.05	14.47	9.95	7.21	16.00	
Housewife	6.82	6.62	7.99	5.47	10.00	
Retired	0.00	0.10	0.00	0.00	0.00	
Free lancer	3.27	3.23	4.52	2.38	0.00	
Jobless	7.09	6.39	10.41	10.95	18.00	
Other	1.23	1.06	1.81	0.75	0.00	
Self-reported health status (%)						<0.0001*
Excellent or good	85.79	81.34	60.00	53.62	59.18	
Fair	3.45	4.93	19.69	5.88	0.00	
Very bad or poor	10.76	13.73	20.31	40.50	40.82	
Urbanization (%)						<0.0001*
Urban	62.06	70.80	75.19	66.29	84.00	
Rural	37.94	29.20	24.81	33.71	16.00	
Regular LTPA (%)						<0.0001*
Yes	30.62	25.55	85.99	82.65	83.67	
No	69.38	74.45	14.01	17.35	16.33	

LTPA, leisure-time physical activity; SD, standard deviation. Values are expressed as means ± SD or %. ^*^p < 0.05.

Table 2 compares intergroup differences by happiness levels and BMI. Significant differences in BMI measurements were observed across all happiness levels, with Tukey’s test revealing that the unhappy group had significantly higher BMI than did the very happy group, while the happy and fair groups had significantly higher BMI than did the very unhappy group. The results also compared the BMI category prevalence between different happiness levels in young adults. The prevalence of BMI categories for all happiness groups was significantly different (*p* < 0.001).

**Table 2:** Differences in BMI means and prevalence of BMI categories by happiness levels in Taiwanese young adults.

Variables	Very happy (*n* = 730)	Happy (*n* = 8,246)	Fair (*n* = 643)	Unhappy (*n* = 795)	Very unhappy (*n* = 50)	*p*	Tukey’s *post hoc* test
BMI (kg/m^2^)	22.86 ± 3.75	23.03 ± 3.87	23.29 ± 4.35	23.68 ± 4.39	20.50 ± 3.31	<0.0001*	U > VH, H, F > VU
BMI categories (%)						<0.0001*	
Obesity	14.01	14.73	18.43	20.61	2.05		
Overweight	20.45	20.30	18.43	19.85	12.24		
Normal weight	56.86	55.96	55.61	52.62	55.10		
Underweight	8.68	9.01	7.53	6.92	30.61		

BMI, body mass index; F, fair; H, happy; SD, standard deviation; U, unhappy; VH, very happy; VU, very unhappy. Values are expressed as means ± SD or %. ^*^p < 0.05.

The results of the multiple linear regression for happiness levels and BMI are shown in Table 3. The happiness levels were significantly and positively associated with BMI in very happy (*β* = 2.363, *p* < 0.001), happy (*β* = 2.529, *p* < 0.001), fair (*β* = 2.792, *p* < 0.001), and unhappy (*β* = 3.177, *p* < 0.001) groups. After adjusting for age, sex, education, occupation, self-reported health status, urbanization of the residential place, and regular LTPA, participants in very happy (*β* = 3.167, *p* < 0.001), happy (*β* = 3.239, *p* < 0.001), fair (*β* = 3.058, *p* < 0.001), and unhappy (*β* = 2.942, *p* < 0.001) groups exhibited a significantly higher BMI than did participants in very unhappy group.

**Table 3:** Associations between happiness levels and BMI among Taiwanese young adults.

Happiness levels	Model 1 (unadjusted)			Model 2 (adjusted^a^)		
	*β*	S.E.	*p*	*β*	S.E.	*p*
Very happy	2.363	0.582	<0.0001*	3.167	0.531	<0.0001*
Happy	2.529	0.564	<0.0001*	3.239	0.515	<0.0001*
Fair	2.792	0.584	<0.0001*	3.058	0.533	<0.0001*
Unhappy	3.177	0.580	<0.0001*	2.942	0.529	<0.0001*
Very unhappy	Ref.	—	—	Ref.	—	—

BMI, body mass index; LTPA, leisure-time physical activity; SE, standard error. Estimates are derived from multiple linear regression models. ^a^Adjusted for age, gender, education, occupation, self-reported health status, urbanization of the residential place, and regular LTPA. *p < 0.05.

Table 4 presents the multivariate adjusted ORs for obesity in relation to happiness levels, after adjusting for potential confounders. The very happy, happy, fair, and unhappy groups showed no statistically significant association not only before adjustment (OR = 2.845, 95% CI: 0.358–22.629; OR = 2.562, 95% CI: 0.327–20.057; OR = 3.567, 95% CI: 0.446–28.498; OR = 3.404, 95% CI: 0.429–27.011) but also after adjusting for confounders (OR = 7.941, 95% CI: 0.906–69.630; OR = 6.331, 95% CI: 0.736–54.437; OR = 6.775, 95% CI: 0.770–59.611; OR = 4.968, 95% CI: 0.570–43.295).

**Table 4:** Associations between happiness levels and obesity.

Happiness levels	Model 1 (unadjusted)			Model 2 (adjusted^a^)		
	OR	95% CI	*p*	OR	95% CI	*p*
Very happy	2.845	0.358–22.629	0.323	7.941	0.906–69.630	0.061
Happy	2.562	0.327–20.057	0.370	6.331	0.736–54.437	0.093
Fair	3.567	0.446–28.498	0.230	6.775	0.770–59.611	0.085
Unhappy	3.404	0.429–27.011	0.246	4.968	0.570–43.295	0.147
Very unhappy	Ref.	—	—	Ref.	—	—

CI, confidence interval; LTPA, leisure-time physical activity; OR, odds ratio. ORs are obtained from logistic regression models. ^a^Adjusted for age, gender, education, occupation, self-reported health status, urbanization of the residential place, and regular LTPA. *p < 0.05.

Table 5 presents the multivariate adjusted ORs for overweight in relation to happiness levels, after adjusting for potential confounders. The very happy, happy, fair, and unhappy groups showed no statistically significant association not only before adjustment (OR = 1.221, 95% CI: 0.328–4.544; OR = 1.187, 95% CI: 0.326–4.324; OR = 1.253, 95% CI: 0.333–4.710; OR = 1.007, 95% CI: 0.270–3.760) but also after adjusting for confounders (OR = 1.776, 95% CI: 0.460–6.861; OR = 1.610, 95% CI: 0.427–6.073; OR = 1.407, 95% CI: 0.361–5.487; OR = 0.997, 95% CI: 0.252–3.777).

**Table 5:** Associations between happiness levels and overweight.

Happiness levels	Model 1 (unadjusted)			Model 2 (adjusted^a^)		
	OR	95% CI	*p*	OR	95% CI	*p*
Very happy	1.221	0.328–4.544	0.766	1.776	0.460–6.861	0.405
Happy	1.187	0.326–4.324	0.795	1.610	0.427–6.073	0.482
Fair	1.253	0.333–4.710	0.739	1.407	0.361–5.487	0.622
Unhappy	1.007	0.270–3.760	0.992	0.977	0.252–3.777	0.973
Very unhappy	Ref.	—	—	Ref.	—	—

CI, confidence interval; LTPA, leisure-time physical activity; OR, odds ratio. ORs are obtained from logistic regression models. ^a^Adjusted for age, gender, education, occupation, self-reported health status, urbanization of the residential place, and regular LTPA. *p < 0.05.

Table 6 presents the multivariate adjusted ORs for underweight in relation to happiness levels, after adjusting for potential confounders. Statistical significance was observed in the very happy, happy, and unhappy groups before adjustment (OR = 0.276, 95% CI: 0.089–0.859; OR = 0.330, 95% CI: 0.112–0.970; OR = 0.264, 95% CI: 0.085–0.822). After adjusting for confounders, the very happy, happy, fair, and unhappy groups showed statistical significance (OR = 0.196, 95% CI: 0.061–0.633; OR = 0.258, 95% CI: 0.085–0.785; OR = 0.271, 95% CI: 0.083–0.882; OR = 0.222, 95% CI: 0.069–0.714).

**Table 6:** Associations between happiness levels and underweight.

Happiness levels	Model 1 (unadjusted)			Model 2 (adjusted^a^)		
	OR	95% CI	*p*	OR	95% CI	*p*
Very happy	0.276	0.089–0.859	0.026*	0.196	0.061-0.633	0.006*
Happy	0.330	0.112-0.970	0.044*	0.258	0.085-0.785	0.017*
Fair	0.331	0.105-1.047	0.060	0.271	0.083–0.882	0.030*
Unhappy	0.264	0.085-0.822	0.022*	0.222	0.069-0.714	0.012*
Very unhappy	Ref.	—	—	Ref.	—	—

CI, confidence interval; LTPA, leisure-time physical activity; OR, odds ratio. ORs are obtained from logistic regression models. ^a^Adjusted for age, gender, education, occupation, self-reported health status, urbanization of the residential place, and regular LTPA. *p < 0.05.

## 4 Discussion

This study aimed to determine the association of happiness levels with BMI and obesity status in Taiwanese young adults aged 18–44 years, adopting a large-scale, representative database to conduct a national cross-sectional study. Although a substantial body of research has examined the relationship between BMI and happiness-related constructs, much of it centers on broader measures such as general wellbeing or life satisfaction, rather than directly assessing happiness as a distinct outcome. Our study advances this literature by focusing specifically on self-reported happiness, offering a more direct assessment of this association. To our knowledge, no prior research has investigated this relationship in the context of Taiwan, particularly among young adults. Given Taiwan’s unique sociocultural and economic landscape—including distinct societal attitudes toward body image and health—our findings offer novel insights that may not be reflected in studies conducted elsewhere. By concentrating on young adults, we also highlight a critical developmental stage in which the interplay between BMI and happiness may be especially salient.

The results of this study revealed a positive and significant association between happiness levels and BMI. Specifically, higher levels of happiness were associated with a lower likelihood of being underweight. In other words, young adults reporting greater happiness tended to have higher BMI compared to their less happy peers. However, the distribution of individuals classified as “very happy” differed significantly across BMI categories, particularly among those who were underweight.

The findings of present study were consistent with those of previous studies, which found that very happy or unhappy groups had a negative association with the underweight group. However, previous studies have indicated that underweight and unhappiness have a negative association. On the other hand, people in different levels with higher or lower body satisfaction could have led to changes in happiness levels (47), as could other potential mechanisms. A previous study found that cultural differences such as lifestyle, food, GDP, and social support can lead to different outcomes from the West in this relationship (48).

Additionally, this is inconsistent with previous studies on adults (49, 50) that reported significantly lower levels of happiness in the obese group. In general, individuals with high BMI, especially women, experience greater social stress to be thin (51, 52). This pressure could lead to multiple forms of prejudice and discrimination (53–55), anxiety (56), negative affect (57), and body dissatisfaction (50, 58, 59). However, previous research has also revealed that excessive weight loss may lead to unhealthy weight control behaviors, which have been found to be associated with an increased risk of developing eating disorders (60). Therefore, a causal relationship between obesity and unhappiness must be explored.

Some limitations of the study should be considered. First, the present study used a cross-sectional design that could not confirm the causal relationship between the variables. Future research should employ longitudinal data to better account for individual-specific factors and assess causal mechanisms. Second, while we controlled for several key confounding variables, unobserved factors such as genetic predispositions or stable personality traits may still influence the association. Alternative approaches, such as longitudinal analyses or instrumental variable methods, could help address these concerns. Third, our study used a national secondary database, and the self-reported nature of the anthropometric data may introduce bias, potentially affecting internal validity.

## 5 Conclusion

Overall, this study revealed that happiness levels were positively associated with BMI, and the increments of happiness levels may have reducing the risk of underweight occurrence. Future studies should emphasize the development and control of body components across all ages to maintain their functional abilities and happiness levels.

## Data Availability

The data that support the findings of this study are available from the Sports Cloud: Information and Application Research Center of Sports for All, Sport Administration, Ministry of Education in Taiwan but restrictions apply to the availability of these data, which were used under license for the current study, and so are not publicly available. Data are however available from the corresponding author upon reasonable request and with permission of the Sports Cloud: Information and Application Research Center of Sports for All, Sport Administration, Ministry of Education in Taiwan.
